# Hydrolyzed protein supplementation improves protein content and peroxidation of skeletal muscle by adjusting the plasma amino acid spectrums in rats after exhaustive swimming exercise: a pilot study

**DOI:** 10.1186/1550-2783-11-5

**Published:** 2014-02-24

**Authors:** Xinying Wang, Chenglin Niu, Jun Lu, Ning Li, Jieshou Li

**Affiliations:** 1 Research Institute of General Surgery, Jinling Hospital, Medical School, Nanjing University, 305 East Zhongshan Road, Nanjing 210002, Jiangsu Province, People’s Republic of China; 2 Department of ICU, Brain Hospital, Nanjing Medical University, 264 Guangzhou Road, Nanjing 210029, Jiangsu Province, China; 3 Department of Orthopedics, Zhongda Hospital, Southeast University, 87 Dingjiaqiao, Nanjing 210009, Jiangsu Province, China

**Keywords:** Protein hydrolysates, Oxidative stress, Amino acid spectrum, Physical training

## Abstract

**Background:**

This study was designed to evaluate the effects of hydrolyzed protein supplementation upon skeletal muscle total protein and peroxidation in rats following exhaustive swimming exercise.

**Methods:**

Twenty-four rats were randomized to 4 experimental groups (n = 6 per group): control group fed standard diet without exercise (SD), exercise (EX), exercise plus standard diet for 72 hours (EX + SD), and exercise plus standard diet supplemented with hydrolyzed protein (2 g/kg/d) for 72 hours (EX + HP). Immediately following exercise, the EX group was euthanized for collecting plasma and skeletal muscle samples. The EX + SD and EX + HP groups were fed their respective diets for 72 hour still plasma and skeletal muscle collection. Skeletal muscle samples were used to measure levels of total protein (TP), malondialdehyde (MDA), and protein carbonyl (PC). Plasma samples were used to analyze the amino acids spectrum.

**Results:**

Compared with the EX + SD, EX + HP presented the significantly increased TP (P = 0.02) and decreased MDA and PC levels (P = 0.035). MDA was negatively correlated with the methionine levels. Moreover, EX + HP maintained higher levels of plasmaleucine, isoleucine, and methionine than EX + SD, which may be associated with the increased skeletal muscle TP levels observed (P < 0.05).

**Conclusions:**

These results collectively suggest that hydrolyzed protein supplementation can improve skeletal muscle TP and ameliorate peroxidation damage in rats subjected to exhaustive exercise stress, which may be, at least in part, related with the maintenance of plasma leucine, isoleucine, and methionine levels.

## Background

Exercise promotes muscle protein turnover, resulting in the specific morphological and metabolic skeletal muscle adaptation [1,2]. Exhaustive exercise leads to myofibrillar degradation and is associated with the decreased force generating capabilities of muscle at fatigue [3]. Muscle protein loss following exhaustive exercise is accompanied by a direct detection of free-radical generation in whole body and skeletal muscle [4,5]. The elevated lipid and protein peroxidation, malondialdehyde (MDA) and protein carbonyl (PC) have been observed in different tissues including skeletal muscle in rats following exhaustive exercise [6,7]. As a result, excessive reactive oxygen species (ROS) can attack the vital biomolecules, such as plasma membrane lipids and proteins, and further deteriorates normal cellular functions and delays recovery from fatigue. Hence, adequate amino acid is required for skeletal muscle to meet the increasing demand of protein retention and reduce the peroxidation following exhaustive exercise. It is beneficial for the fast recovery from athletes during competition season. However, promoting positive muscle protein balance is dependent upon the availability of nutrient metabolites and the lack of appropriate nutrient intake can lead to a net negative protein balance and ROS accumulation [8,9]. This loss leads to a decrease in muscular strength, delayed recovery from fatigue, and decreased resistance to stress (disease or trauma) [3].

Previous studies suggest that standard diets cannot supply enough nutrients after exercise due to metabolic derangement in tissues [10,11]. Exogenous nutrients or nutritional supplements may help to improve muscle protein content and counteract the oxidative stress of exercise in subjects unaccustomed to physical activity [12-14]. Supplementations provide a nonpharmacological therapy, and has been gradually received attention in literatures. Protein hydrolysates can stimulate protein synthesis and inhibit protein breakdown, and therefore, improve the net muscle protein balance after exercise [10,15]. It is also reported that whey protein hydrolysate can ameliorate drug-induced oxidative stress [16]. However, it remains to be elucidated whether the protein hydrolysates supplementation in a short term improves the protein retention and oxidative stress of skeletal muscle following exhaustive exercise. Therefore, we hypothesized that an additional hydrolyzed protein supplementation could enhance the muscle protein content and eliminate the oxidative stress products by regulating the plasma amino acid spectrums in rats following exhaustive exercise.

## Methods

### Experimental design

Rats were randomly divided into four groups (n = 6 per group): a control group fed standard diet without exercise (SD), exercise (EX), exercise plus standard diet for 72 h (EX + SD), or exercise plus standard diet supplemented with hydrolyzed protein (2 g/kg/d) for 72 h (EX + HP). Animals were maintained in individual cages and fed a standard chow diet and water *ad libitum*. All rats of the EX, EX + SD and EX + HP groups received a single bout of exhaustive swimming on the first day in the experimental period (time 0 hour). EX was sacrificed immediately following exercise. The animals of the other groups had open access to a standard rodent chow diet and water *ad libitum* throughout the study. A standard lab rat diet was rich in dietary fiber, trace elements, and intact protein (18 g/100 g fodder) including 1.76 g leucine and 5 g crude fiber per 100 g fodder.

Additionally, the EX + HP group received a supplementation of protein hydrolysate (6.67 ml/kg body weight) by oral gavage once per day, while EX + SD received the same value of purified water via oral gavage. The protein hydrolysates (HYDROPROTEIN, Shen Yi Food Nutrition, Zhuji, ZJ.) contain 60% hydrolyzed whey protein as its source of nitrogen, providing a rich source of leucine (4.67 g/100 g powder) (powder, 50 g/per bag). The protein consists of 100% content of di- and tripeptides. It was dissolved in purified water (Nestle Company, USA) and the final protein concentration was 0.3 g/ml. After 72 hours of feeding following exercise, both EX + SD and EX + HP groups were sacrificed for sample collection.

### Subjects

Twenty-four 7-week-old (250 g) specific pathogen-free male Sprague Dawley male rats were used and individually housed in a metabolic cage at the Jinling hospital Animal Research facility at Nanjing, Jiangsu province. They were placed in a room maintained at 22°C with a 12: 12-hour light: dark cycle and provided with rodent chow and water *ad libitum.* This experimental study was approved and performed in strict accordance with the guidelines for the Institutional Animal Care and Use committee, Jinling hospital trial registration (2010NKY062). The experimental model was conducted in a manner consistent with the relevant ethical guidelines for animal research, Jinling hospital. All surgery was performed under pentobarbital anesthesia, and all efforts were made to minimize suffering.

### Exhaustive exercise model

We chose the swimming model as an exhaustive physical training model. The rats were hanging a heavy object which accounted for 3% of their weight, then were placed into a 40 cm × 40 cm × 100 cm container filled with water (30°C) [17]. In our preliminary test, we examined the swimming time period and the appropriate load weight of swimming rats. It was found that rats would float if the hanging weight was lower than 3% of body weight and would easily sink if it was more than 6% of body weight. So we chose the 3% of body weight as load weight tied to their tails. Animals were removed from the swimming chamber when they were exhausted, as determined by their inability to surface after repeated attempts, or their remaining below the water surface for 10 s. The average swimming time was is about 140 min in the rat model. And so the exercise intensity was similar among the three groups. The rats were wiped up by dry and warm towels in the warm room to prevent the thermoregulatory response.

### Procedures

The rats were anesthetized with pentobarbital (50 mg/kg body weight). Blood was rapidly collected from the abdominal aorta and plasma was immediately separated after centrifugation at 5000 g for 5 minutes (Ningbo Hinotek Technology Co., Ltd., China) at 4°C, then placed in -80°C until assay. The gastrocnemius was removed and washed in 0.9% cold saline and placed immediately in liquid nitrogen.

#### ***Body weight, food intake and excrement measurement***

All rats were weighed before and after experiment with electronic scale (Furi FEJ-2000B, Shenzhen, China) and the body weight was recorded. Daily food intake and excrement were also recorded.

#### ***Tissue preparation for total protein, MDA and PC determination***

To carry out the assays, the gastrocnemius was weighed and homogenized by adding a 9 times of the volume of 0.9% saline. The 10% homogenate was centrifuged for 10 minutes (1800 g/min) and the supernatant was diluted with 10 times of the volume of 0.9% saline to 1% concentration. All procedures were done in accordance with the manufacturer’s instructions. The 1% supernatant was assayed spectrophotometrically for total protein (TP), malondialdehyde (MDA) and protein carbonyl (PC) activity level with commercial kits (A045-2, A003-1, A087, respectively, Nanjing Jiancheng Bio-engineering Institute, Nanjing, China).

#### ***Analyses of plasma amino acid spectrum***

The plasma amino acids spectrum was quantified by high performance liquid chromatography (HPLC) (Waters 2695, MA, USA). Sample extracts were chromatographed on a column that was kept at 85°C and monitored by fluorescence-detection. H_2_O was used in the mobile phase at a flow-rate of 1.0 ml/min. In brief, 20 μL plasma was mixed uniformly with 100 μL derivative regent (containing phenylisothiocyanate, triethylamine, dehydrated alcohol, deionized water) after thawing, and 20 μL mixed liquid was injected into HPLC pump to measure the plasma concentrations of amino acids. The measurement for all plasma samples were repeated in triplicate [18].

### Statistical analyses

The data are presented as means ± SEM. SPSS16.0 software was applied for statistical analysis of all data (SPPS Inc., Chicago, IL, USA). Differences between groups were examined for statistical significance using one-way analysis of variance (ANOVA) and then determined with the Student-Newman-Keuls test. The correlation was determined by stepwise multiple linear regression. The criterion for significance was P < 0.05.

## Results

### Food intake, excrement and body weight

Groups EX + SD and EX + HP consumed 30 grams of standard diet daily. No significant differences in food intake were observed between groups (SD: 31.0 ± 2.5 g, EX: 33.0 ± 3.1 g, EX + SD: 30.0 ± 1.9 g, EX + HP: 32.0 ± 2.8 g), suggesting protein supplementation did not influence food intake within the 72 hours period. Supplementation of protein hydrolysate or water did not increase the frequency of diarrhea in the EX + SD group and EX + HP group, compared with SD group during the duration of the study (SD: 2.2 ± 0.5 g, EX + SD: 2.5 ± 0.8 g, EX + HP: 2.8 ± 0.6 g).

Before the experiment, there was no difference in body weight among the four groups (SD: 255.7 ± 14.4 g, EX: 265.5 ± 8.5 g, EX + SD: 257.3 ± 8.1 g, EX + HP: 259.7 ± 23.7 g). Following exhaustive swimming exercise, body weights of EX group, EX + SD group and EX + HP group were significantly decreased compared with their initial body weights (EX: 257.5 ± 9.2 g, EX + SD: 253.5 ± 6.4 g, EX + HP: 252.7 ± 19.6 g). At 72 hours after feeding, the body weights of EX + SD group and EX + HP group were higher than immediately following exercise (P < 0.05). The body weight increase observed in EX + HP group was higher compared with EX + SD group (269.7 ± 29.0 g vs 263.0 ± 7.8 g), but the difference did not reach significance (P > 0.05).

### Total protein, PC and MDA levels in rat skeletal muscle

**Figure 1:**
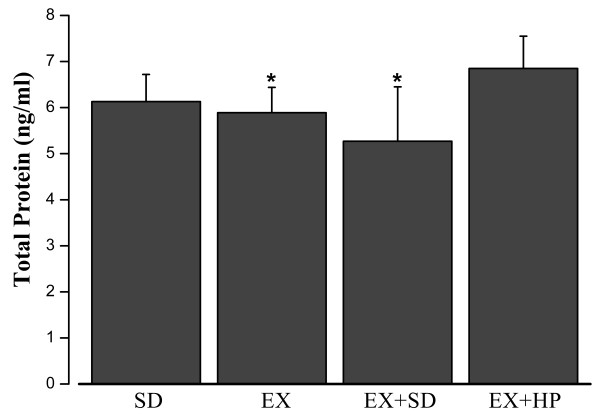
**Concentrations of skeletal muscle total protein levels in standard diet (SD), exercise (EX), exercise plus standard diet for 72 hours (EX + SD), and exercise plus standard diet supplemented with hydrolyzed protein (2 g/kg/d) for 72 hours (EX + HP).** EX + HP group presented significantly higher values than did the EX + SD and EX groups. *P < 0.05: Different from the EX + HP group.

**Figure 2:**
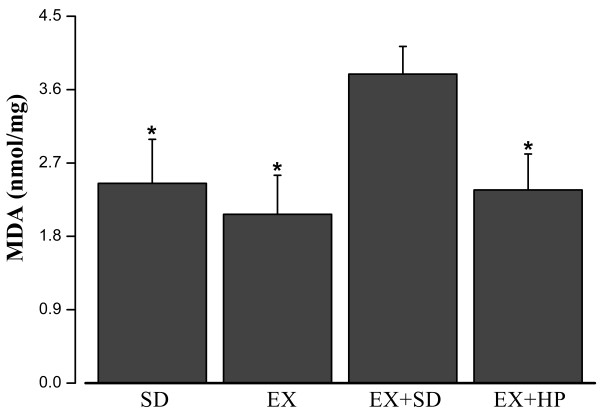
**Concentrations of skeletal muscle malondialdehyde (MDA) levels in standard diet (SD), exercise (EX), exercise plus standard diet for 72 hours (EX + SD), and exercise plus standard diet supplemented with hydrolyzed protein (2 g/kg/d) for 72 hours (EX + HP).** SD, EX and EX + HP groups presented significantly lower values than did the EX + SD group. *P < 0.05: Different from the EX + SD group.

**Figure 3:**
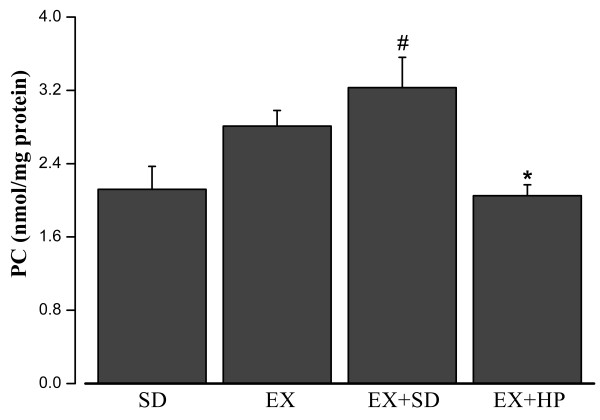
**Concentrations of skeletal muscle protein carbonyl (PC) levels in standard diet (SD), exercise (EX), exercise plus standard diet for 72 hours (EX + SD), and exercise plus standard diet supplemented with hydrolyzed protein (2 g/kg/d) for 72 hours (EX + HP).** EX + HP group presented significantly lower values than did the EX and EX + SD groups. EX + SD group presented significantly higher values than did the SD group.* P < 0.001: Different from the EX and EX + SD groups. # P < 0.001: Different from the SD group.

As illustrated in Figure 1, the total protein amount of skeletal muscle was significantly increased in EX + HP group, compared with EX + SD group (P = 0.02). The level of MDA was significantly lower in EX + HP group compared with EX + SD group (P = 0.035), meanwhile it was elevated in EX + SD group compared with EX group (P = 0.014) (Figure 2). The mean level of PC was increased in EX + SD group compared with SD group (p < 0.001), but it was ameliorated significantly in EX + HP group compared with EX + SD group (p < 0.001) (Figure 3).

### Plasma concentrations of amino acids

The plasma levels of leucine, methionine, phenylalanine, histidine, threonine, arginine, lysine, glycine, valine, serine and cysteine were significantly higher following exercise, compared with SD group (p < 0.05, Table 1). Conversely, the plasma concentration of isoleucine significantly declined in EX + SD during the 72 hours recovery period, compared with groups SD and EX (P < 0.001). Meanwhile, the concentrations of leucine (P = 0.049), isoleucine (P < 0.01) and methionine (P = 0.046) were significantly increased in group EX + HP, compared with group EX + SD. Moreover, there were significant positive correlations between total protein content and leucine (r = 0.993, P < 0.001), isoleucine (r = 0.945, P = 0.004) and methionine (r = 0.902, P = 0.014) levels. Furthermore, significant negative correlation was found between plasma methionine concentration and MDA levels (r = 0.59, P = 0.02) (Table 1).

**Table 1:** The concentrations of plasma free amino acids (AA) of the rats among the standard diet group (SD), exercise group (EX), exercise plus standard diet for 72 h group (EX + SD), and exercise plus standard diet supplemented with hydrolyzed protein (2 g/kg/d) for 72 h group (EX + HP)

**AA (uM)**	**SD**	**EX**	**EX + SD**	**EX + HP**
Aspartic acid	0.146 ± 0.150	0.204 ± 0.061	0.141 ± 0.026	0.127 ± 0.140
Glutamate	0.398 ± 0.126	0.399 ± 0.114	0.283 ± 0.050	0.303 ± 0.036
Serine	0.764 ± 0.131	1.499 ± 0.221^*^	0.861 ± 0.285	0.938 ± 0.177
Glycine	0.960 ± 0.292	1.815 ± 0.176^*^	1.037 ± 0.298	1.112 ± 0.359
Histidine	0.259 ± 0.041	0.519 ± 0.033^*^	0.241 ± 0.057	0.263 ± 0.032
Threonine	0.894 ± 0.298	2.398 ± 0.405^*^	0.668 ± 0.148	1.239 ± 0.708
Alanine	2.092 ± 0.372	2.167 ± 0.343	1.651 ± 0.403	1.990 ± 0.356
Arginine	0.578 ± 0.101	0.924 ± 0.071^*^	0.509 ± 0.122	0.539 ± 0.183
Proline	0.835 ± 0.271	1.035 ± 0.077	0.601 ± 0.030	0.754 ± 0.199
Tyrosine	0.144 ± 0.038	0.177 ± 0.252	0.139 ± 0.063	0.134 ± 0.101
Valine	0.175 ± 0.079	0.923 ± 0.770^*^	0.350 ± 0.062	0.397 ± 0.077^#^
Methionine	0.132 ± 0.019	0.335 ± 0.017^*^	0.081 ± 0.028	0.127 ± 0.041&
Cysteine	1.158 ± 0.083	1.582 ± 0.306^*^	1.204 ± 0.130	1.242 ± 0.047
Isoleucine	0.359 ± 0.018&	0.450 ± 0.136	0.172 ± 0.042^#^	0.368 ± 0.031&
Leucine	0.340 ± 0.190	1.533 ± 0.195^*^	0.284 ± 0.056	0.365 ± 0.070&
Phenylalanine	0.229 ± 0.032	0.507 ± 0.059^*^	0.206 ± 0.015	0.223 ± 0.042
Lysine	1.459 ± 0.443	4.466 ± 0.361^*^	1.251 ± 0.135	1.311 ± 0.405

Note: ^*^P < 0.05 significantly increased compared with SD group; ^#^P < 0.05 significantly decreased compared with SD group; & P < 0.05 significantly increased compared with EX + SD group.

## Discussion

The purpose of this study was to investigate whether hydrolyzed protein supplementation, in a short term, could improve the protein retention and eliminate peroxidation products of skeletal muscle in rats following exhaustive exercise. Our results showed that the protein hydrolysate supplementation improved skeletal muscle protein content and reduced oxidative stress following exhaustive swimming.

Following exhaustive swimming exercise, body weights were dramatically decreased for reasons that were likely multivariable. Acute high intensity swimming can result in energy substrate exhaustion with hepatic glycogen mobilization and skeletal muscle protein catabolism. In addition, catabolism produces water, which is lost during exercise through the skin, respiratory tract and urinary system, to maintain metabolic balance and regulate body temperature. In the present study, there were significant increases in body weight for groups EX + SD and EX + HP after 72 h of feeding, implicating these changes following exercise were temporary and could been restored after post-exercise feeding.

Exercise modifies protein and amino acid metabolism, which is reflected from altered plasma amino acid concentrations [19,20]. Our data demonstrate the levels of leucine, valine, methionine, phenylalanine, histidine, threonine, arginine and lysine were significantly elevated in rats immediately following exhaustive swimming compared with non-exercised controls. It was reported that the increase of plasma amino acid concentrations, particularly leucine and essential amino acids, could activate the key signaling proteins to accelerate the protein anabolism [21-23]. However, significantly reduced levels of leucine, isoleucine, methionine, histidine, threonine, arginine, lysine, glutamate and alanine were observed after 72 hours of recovery and standard diet feeding, which suggest standard diet was insufficient to restore these amino acid levels following exhaustive exercise. In contrast, hydrolyzed protein supplementation not only elevated the levels of leucine, isoleucine and methionine, but also augmented the skeletal muscle protein retention compared with standard diet. Moreover, there were significant positive correlations between the total protein content and leucine, isoleucine and methionine levels. It indicates that the improvement of protein content in skeletal muscle may be a consequence of enhanced plasma leucine, isoleucine and methionine levels following protein hydrolysate supplementation. The present study provides the first evidence that following exhaustive swimming exercise, protein retention was more efficiently improved by supplementation of additional hydrolyzed protein administered in a short term, compared with feeding a standard diet alone in rats.

MDA is suggested to be a biomarker of oxidative stress associated with tissue injury. In addition to MDA, PC may serve as a biomarker of oxidative stress because the oxidation process may be accelerated by the formation and accumulation of carbonylated protein [24,25]. In the present study, a higher level of MDA and PC appeared in rats at 72 hours after exercise, suggesting oxidative stress persists for up to 72 hours following exhaustive exercise. Exercise induced oxidative damage may lead to protein denaturation and loss of essential biological, which causes muscle damage and decreased muscle performance [26,27]. Nutrients can regulate oxidative stress and prevent muscular damage [12,28]. Supplementation of hydrolyzed protein was found to accompany with the reduction of MDA and PC levels, indicating that protein hydrolysate ingestion might ameliorate the peroxidation products of skeletal muscle following exhaustive exercise.

It has demonstrated that methionine, which is distinct from other amino acids, plays a significant role in controlling oxidative stress [29]. In our study, significant negative correlation between plasma methionine concentration and MDA levels was observed. The higher content of methionine (14.2 μg/mg) in our protein hydrolysate might represent a possible mechanism through which hydrolyzed protein supplementation reduces peroxidation damage. In addition, amino acid, especially leucine, was demonstrated to stimulate insulin secretion [30]. An emerging body of evidence suggests that insulin can suppress the inflammatory process through modulating key inflammatory molecules in addition to acting as an anabolic hormone [31]. It thus can be speculated that insulin secretion after feeding with protein hydrolysate may have been responsible, at least in part, for the increased muscle protein retention and improved oxidative stress in rats following exhaust exercise in the present study; however, it needs to be further explored.

Limitations of the current study included a lack of muscle biopsy and morphological assay for structural alterations. Furthermore, measuring plasma amino acid concentration does not provide a measure of the digestion and absorption kinetics for ingested dietary protein. For this reason, we chose the standard diet fed rats as the control to compare the discrimination of amino acid concentrations following 72 hours of post-exercise feeding. Our future research plans include the use of labeled amino acids to accurately assess the appearance rate of amino acids derived from dietary protein in plasma and skeletal muscle.

## Conclusions

In conclusion, the short-term oral supplementation of hydrolyzed protein to standard diet may be an efficacious option in improving protein retention and eliminating reactive oxygen species in rats following exhaustive exercise. Our findings strengthen the importance of protein hydrolysate supplementation in exhaustive exercise-stress situations.

## Abbreviations

SD: Standard diet without exercise; EX: Exercise; EX + SD: Exercise plus standard diet; EX + HP: Exercise plus standard diet supplemented with hydrolyzed protein; TP: Total protein; MDA: Malondialdehyde; PC: Protein carbonyl; ROS: Reactive oxygen species; HPLC: High performance liquid chromatography.

## Competing interests

The authors declare that they have no competing interests.

## Authors’ contributions

XW and CN carried out the animal studies and participated in the samples measurement. XW drafted the manuscript. JL performed the statistical analysis and helped to draft the manuscript. NL and JL sconceived of the study, and participated in its design and coordination. All authors read and approved the final manuscript.
